# Dapagliflozin, a Sodium-Glucose Co-Transporter 2 Inhibitor, Acutely Reduces Energy Expenditure in BAT via Neural Signals in Mice

**DOI:** 10.1371/journal.pone.0150756

**Published:** 2016-03-10

**Authors:** Yumiko Chiba, Tetsuya Yamada, Sohei Tsukita, Kei Takahashi, Yuichiro Munakata, Yuta Shirai, Shinjiro Kodama, Yoichiro Asai, Takashi Sugisawa, Kenji Uno, Shojiro Sawada, Junta Imai, Kazuhiro Nakamura, Hideki Katagiri

**Affiliations:** 1 Department of Metabolism and Diabetes, Tohoku University Graduate School of Medicine, Sendai, 980-8575, Japan; 2 Department of Integrative Physiology, Nagoya University Graduate School of Medicine, Nagoya, 466-8550, Japan; 3 Japan Agency for Medical Research and Development (AMED), CREST, Sendai, 980-8575, Japan; University of Minnesota, UNITED STATES

## Abstract

Selective sodium glucose cotransporter-2 inhibitor (SGLT2i) treatment promotes urinary glucose excretion, thereby reducing blood glucose as well as body weight. However, only limited body weight reductions are achieved with SGLT2i treatment. Hyperphagia is reportedly one of the causes of this limited weight loss. However, the effects of SGLT2i treatment on systemic energy expenditure have not been fully elucidated. Herein, we investigated the acute effects of dapagliflozin, a SGLT2i, on systemic energy expenditure in mice. Eighteen hours after dapagliflozin treatment oxygen consumption and brown adipose tissue (BAT) expression of *ucp1*, a thermogenesis-related gene, were significantly decreased as compared to those after vehicle treatment. In addition, dapagliflozin significantly suppressed norepinephrine (NE) turnover in BAT and *c-fos* expression in the rostral raphe pallidus nucleus (rRPa) which contains the sympathetic premotor neurons responsible for thermogenesis. These findings indicate that the dapagliflozin-mediated acute decrease in energy expenditure involves a reduction in BAT thermogenesis via decreased sympathetic nerve activity from the rRPa. Furthermore, common hepatic branch vagotomy abolished the reductions in *ucp1* expression and NE contents in BAT and *c-fos* expression in the rRPa. In addition, alterations in hepatic carbohydrate metabolism, such as decreases in glycogen contents and upregulation of phosphoenolpyruvate carboxykinase, manifested prior to the suppression of BAT thermogenesis, e.g. 6 hours after dapagliflozin treatment. Collectively, these results suggest that SGLT2i treatment acutely suppresses energy expenditure in BAT via regulation of an inter-organ neural network consisting of the common hepatic vagal branch and sympathetic nerves.

## Introduction

The number of obese patients is tremendously increasing at an alarming rate not only in the industrialized nations but also in developing countries. Obesity is a risk factor for type 2 diabetes (T2D), hypertension and dyslipidemia. These disorders are related to serious health problems such as cardio- and cerebro-vascular diseases. Therefore, the development of therapeutic strategies for T2D that include decreasing body weight has long been awaited. Most drugs for T2D enhance insulin secretion and/or improve insulin sensitivity. Recently, the sodium glucose cotransporter-2 inhibitors (SGLT2is) were recently developed as a novel class of drugs for T2D [1]. Sodium glucose cotransporter-2 (SGLT2) is expressed on the luminal surface of renal proximal tubular cells, and accounts for approximately 90% of renal glucose reabsorption [2]. Effects on increased urinary glucose excretion (UGE) by selective SGLT2 inhibition are not limited to blood glucose reduction in an insulin-independent manner but body weight reduction is also achieved [3]. However, weight loss degrees after SGLT2i treatment appear to be much less than those predicted from the excreted glucose amounts in both human and animal studies [4,5]. One of the reasons for this limitation is reportedly the induction of hyperphagia in rodents and humans [6–8]. The energy loss due to the increased UGE may cause hyperphagia in order to compensate for a negative energy balance. Consistently, pair-fed SGLT2i-treated rats, which were given the same amounts of food as those consumed by vehicle controls (SGLT2i-PF rats), showed approximately 4-fold greater weight loss than their counterparts who were allowed ad libitum access to food after SGLT2i treatment [6]. However, hyperphagia cannot fully explain the limited body weight reductions observed. For instance, body weight loss in SGLT2i-PF rats decreased by only -13.2% and did not further decrease despite ongoing substantial glucose excretion [6]. These findings prompted us to hypothesize that SGLT2i suppresses systemic energy expenditure. In this study, we examined whether SGLT2i treatment actually suppresses energy expenditure and, if so, to identify the mechanism(s) involved using an experimental model of food deprivation for 24h after dapagliflozin administration.

## Materials and Methods

### Animals

Eight-week-old male C57BL/6 mice (CLEA Japan, Inc. Tokyo, Japan) were individually housed under specific-pathogen free conditions with controlled temperature (25°C) and a 12-h/12-h light/dark cycle. The mice had access to the standard laboratory diet (65% carbohydrate, 4% fat, 24% protein) and water *ad libitum* unless otherwise noted. The mice were sacrificed by cervical dislocation. Animal studies were conducted in accordance with Tohoku University institutional guidelines. All of the experimental protocols had been approved by the Institutional Animal Care and Use Committee of the Tohoku University Environmental and Safety Committee prior to undertaking these experiments.

### Chemicals

Dapagliflozin ((1S)-1, 5-anhydro-1-C-[4-chloro-3-[(4-ethoxyphenyl) methyl] phenyl]-D-glucitol) was synthesized by Cayman Chemical (MI, USA) at an estimated purity of >98%.

### Design

**Fig 1:**
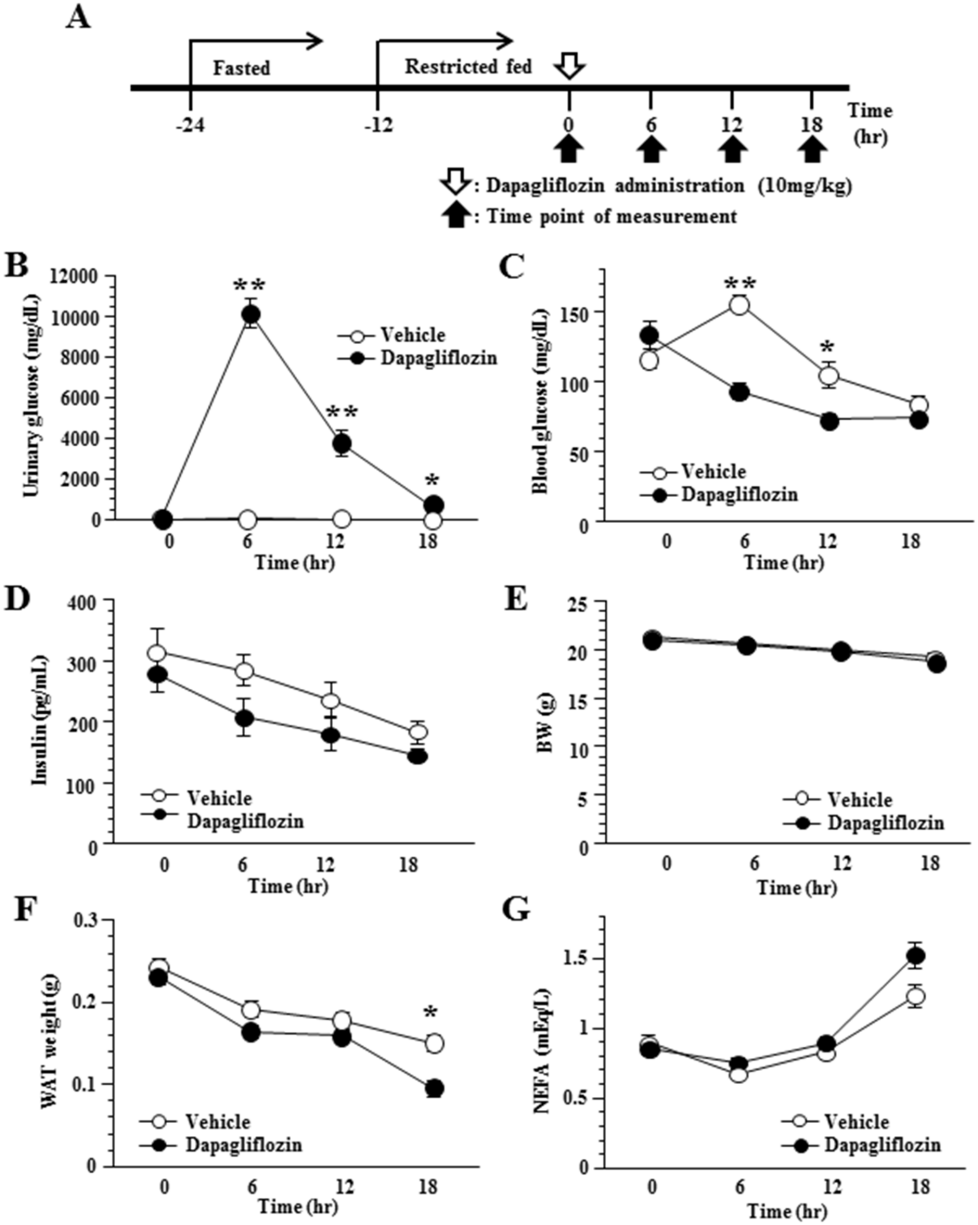
Administration of dapagliflozin increased UGE and reduced blood glucose levels. Timeline of study design (A). Urinary glucose (B) and plasma blood glucose (C) after a single oral dose of dapagliflozin (10mg/kg). Plasma insulin (D), body weight (BW) (E), WAT weight (F) and plasma NEFA (G) in SGLT2i- and control-mice. Values shown are means ±SEM (n = 6–7 per group). Significance as compared to control-mice is indicated (**P < 0.01 and *P < 0.05).

After being acclimated to 25°C chambers for 7 days, the mice were assigned to two treatment groups based on body weight before dapagliflozin administration. In order to minimize the individual differences in energy conditions among mice at baseline, starting from 24h before drug treatment, the mice were fasted for 12h, which was followed by an additional 12h of restricted chow diet (5.4kcal) (Fig 1A). Dapagliflozin (10mg/kg) or vehicle (0.5% carboxymethylcellulose sodium salt (CMC); 20ml/kg) was delivered to the mice by single oral gavage. Four independent groups of mice corresponding to different sacrifice time points were prepared.

### Urinary glucose and blood analysis

Urine samples were collected from mice before or 6, 12 or 18h after dapagliflozin administration. Urinary glucose concentrations were analyzed by Oriental Yeast Co., Ltd, (Tokyo, Japan). Blood glucose levels were assayed with Glutestmint (Sanwa Kagaku Kenkyusho CO., LTD. Aichi, Japan). Plasma samples were collected from mice before or 6, 12 or 18h after dapagliflozin administration. Plasma insulin and leptin levels were determined using an ELISA kit (Morinaga Institute of Biological Science, Yokohama, Japan). Plasma non-esterified fatty acid (NEFA) concentrations were determined with a NEFA C kit (Wako Pure Chemical Co., Osaka, Japan). Plasma glucagon was analyzed by SRL (Tokyo, Japan), and plasma norepinephrine and epinephrine by BML (Tokyo, Japan).

### Oxygen consumption and respiratory exchange rate

Mice were acclimated to 25°C metabolic chambers for 7 consecutive days, and were then administered dapagliflozin by single oral gavage. Oxygen consumption was determined with an O_2_/CO_2_ metabolism measuring system for 24h (MK-5000RQ, Muromachikikai, Tokyo, Japan).

### Measurement of BAT and rectal temperature

After the mice had been anesthetized, a small area of skin above the BAT was surgically removed leaving the underlying tissue intact. A needle microprobe (Thermalert MT-290/1, Physitemp, NJ, USA) was inserted through the tissue membrane into BAT, and the temperature was thereby measured. Rectal temperature was measured with a rectal probe (Thermalert TH-5, Physitemp).

### NE turnover

NE turnover was measured on the basis of the decline in tissue NE content after the inhibition of catecholamine biosynthesis with α-methyl-p-tyrosine (α-MT) (Santa Cruz Biotechnology, Inc, TX, USA). Eighteen hours later the mice were administrated dapagliflozin or vehicle and then injected with α-MT (200mg/kg, i.p.). At 0 and 4h after α-MT injection, the mice were sacrificed by cervical dislocation and tissues were rapidly removed and weighed. Tissue samples were homogenized in 0.4M HClO. The homogenates were centrifuged at 4°C, and the NE content of the resulting supernatants was measured by SRL (Tokyo, Japan).

### Immunohistochemical detection of c-fos protein

The mice were anesthetized and transcardially perfused with 2% paraformaldehyde. The brain was removed, immediately postfixed, embedded in OCT compound, and the appropriate brain area was then sectioned into 30μm slices. The sections were rinsed in TBS-T, and endogenous peroxidase was blocked by 3% H_2_O_2_ in PBS. The sections were incubated for 72h at 4°C with c-fos antibody (#2250, Cell Signaling Technology, MA, USA) diluted with Can Get Signal Immunostain Solution B (TOYOBO, Osaka, Japan) at a dilution rate of 1:3000. After washing, the sections were incubated with Simplestain MAX-PO (R) (Nichirei, Tokyo, Japan) for 60 min at room temperature followed by incubation with 0.05% DAB solution for 10 min to make c-fos visible. Sections were stained with hematoxylin, followed by dehydration, and were then mounted employing a coverslip. Sections were analyzed qualitatively with a light microscope. c-fos-positive cells in six consecutive sections of the rRPa were counted in each mouse, and the mean number of counts in each group of mice was determined.

### Laser microdissection

Coronal cryostat sections (25μm) of the rostal raphe pallidus nucleus (rRPa) were placed on PEN-coated slides (Leica Microsystems). Laser microdissection was carried out on a Leica AS LMD (Leica Microsystems). Immediately after microdissection, total RNA was purified as previously described [9].

### Measurement of liver glycogen content

Liver glycogen contents were measured with a Glycogen Assay Kit (Bio Vision, CA, USA).

### Histological analysis

Livers were removed and fixed with 10% formalin and embedded in paraffin. Tissue sections were stained with periodic acid-Schiff.

### Dissection of the common hepatic branch of the vagal nerve

Mice were subjected to either common hepatic branch vagotomy (CHBV) or sham operation (SO), as previously described [10,11]. After being anesthetized with a combination of three anesthetics: medetomidine hydrochloride (Domitol, Meiji Seika Pharma Co.,Ltd., Tokyo, Japan) 0.3mg/kg; midazolam (Dormicum, Astellas Pharma Inc., Tokyo, Japan) 4mg/kg; and butorphanol (Vetorphale, Meiji Seika Pharma Co.,Ltd.) 5mg/kg), a laparotomy incision was made on the ventral midline followed by a second incision to open the abdominal muscle wall. The gastrohepatic ligament was severed using fine forceps, and the stomach was gently retracted, revealing the descending ventral esophagus and the ventral subdiaphragmatic vagal trunk. The common hepatic branch of the vagal trunk was then transected using fine forceps. All mice were carefully monitored every 4 hours for 24 hours during the postoperative recovery period to detect any events indicative of dyspnea, hypothermia, ataxia or other forms of physiological dysfunction.

### Statistical analysis

All data are expressed as means ± SEM. The statistical significance of differences was assessed using either the two-tailed unpaired t-test, one-way ANOVA or two-way ANOVA followed by Tukey’s post hoc test, as appropriate.

## Results

### Administration of dapagliflozin increased UGE and reduced blood glucose levels in mice

First, we examined the effects of dapagliflozin, a SGLT2 inhibitor, on glucose and energy metabolism in 8-week-old C57BL/6 mice. In this experiment, to exclude the effects of food intake on energy expenditure, the mice were housed without food after dapagliflozin administration. Oral administration of a single dose of dapagliflozin (10mg/kg) caused a marked increase in urinary glucose in these mice (SGLT2i-mice) which lasted for 18 hours (Fig 1B). SGLT2i-mice had significantly lower blood glucose levels than vehicle-treated mice (control-mice) 6 and 12 hours after administration (Fig 1C). There were no significant differences in plasma insulin levels between SGLT2i- and control-mice (Fig 1D). Under these conditions, body weights did not differ significantly between SGLT2i- and control-mice throughout the 18-hour period after administration (Fig 1E). White adipose tissue (WAT) weights were slightly decreased after 18 hours (Fig 1F). However, plasma NEFA levels were similar (Fig 1G) in SGLT2i- and control-mice throughout the experimental period, suggesting that lipolysis was similarly induced in these two groups. Overall, despite energy loss as UGE in SGLT2i-mice, their body weights were similar to those of control-mice.

### Dapagliflozin acutely suppressed BAT thermogenesis by reducing sympathetic nerve activity in mice

**Fig 2:**
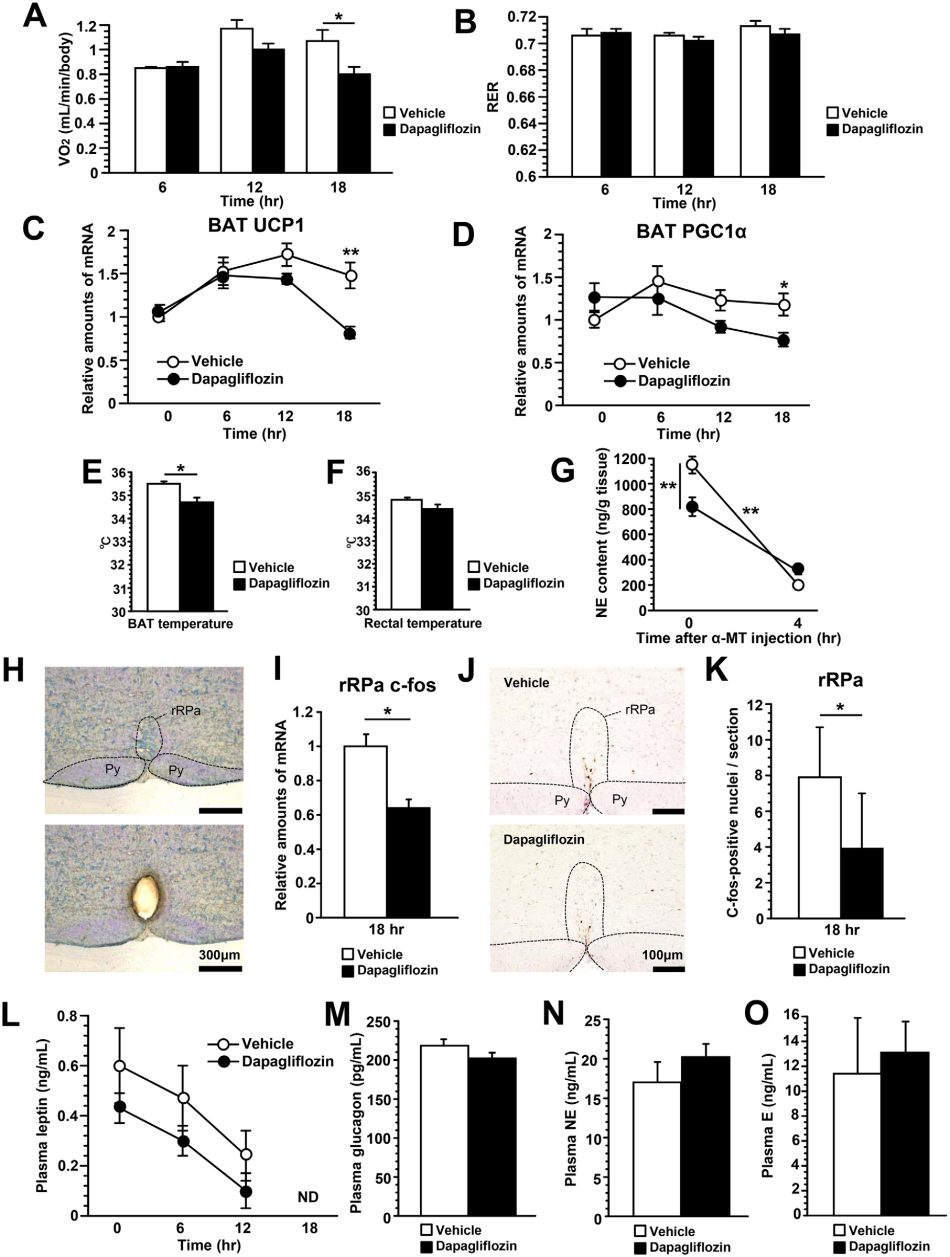
Dapagliflozin acutely suppressed BAT thermogenesis by reducing sympathetic nerve activity. Oxygen consumption during the 18 hours following dapagliflozin administration (A). Respiratory exchange rate (RER) during the 18 hours following dapagliflozin administration (B). Relative amounts of UCP1 (C) and PGC1α (D) mRNA in BAT. BAT (E) and rectal (F) temperatures 18 hours after dapagliflozin administration. NE turnover in BAT 18 hours after dapagliflozin administration (G). The difference in the NE decline between SGLT2i- and control-mice due to α-methyl-*p*-tyrosine (α-MT) treatment was statistically analyzed employing two-way ANOVA. Coronal section of the rostral ventromedial medulla before (upper panel) and after (lower panel) microdissection of the rRPa. Py, pyramidal tract (H). Relative amounts of c-fos mRNA in the rRPa 18 hours after dapagliflozin administration (I). Immunohistochemical detection of c-fos protein in the rRPa (J). The number of c-fos positive neurons in the rRPa (K). Plasma leptin levels during the 18 hours following dapagliflozin administration (L). Plasma glucagon (M), NE (N) and epinephrine (E) (O) levels 18 hours after dapagliflozin administration. Values shown are means ±SEM (n = 6–7 per group). Significance as compared to control-mice is indicated (**P < 0.01 and *P < 0.05 and). ND = not detectable.

Next, to evaluate systemic energy expenditure, we examined oxygen consumptions of SGLT2i- and control-mice. Eighteen hours after dapagliflozin treatment, oxygen consumption was significantly reduced in SGLT2i-mice (Fig 2A). Respiratory exchange rates (RER) were similar in these mice at almost 0.7 during the 18 hours (Fig 2B), suggesting this observation to reflect food deprivation. BAT thermogenesis also plays an important role in whole-body energy expenditure [12]. Interestingly, expressions of thermogenesis-related genes, such as uncoupling protein-1 (*UCP1*) (Fig 2C) and peroxisome proliferator activated receptor-gamma coactivator 1 α (*PGC-1α*) (Fig 2D), were lower in BAT of SGLT2i-mice than in that of control-mice 18 hours after dapagliflozin administration. Consistent with these results, BAT temperature 18 hours after dapagliflozin administration was significantly decreased in SGLT2i-mice as compared to control-mice (Fig 2E). In addition, rectal temperature of SGLT2i-mice tended to be lower than that of control-mice (Fig 2F). Taken together, these results indicate that the energy expenditure suppression caused by acute dapagliflozin treatment involves reduced BAT thermogenesis.

Activation of sympathetic nerves to BAT is responsible for *UCP1* and *PGC1α* up-regulation, followed by enhancement of BAT thermogenesis [13]. Therefore, we first examined norepinephrine (NE) turnover in BAT, as this result is known to reflect sympathetic activity to BAT [14]. NE contents in BAT 18 hours after dapagliflozin administration were significantly suppressed in SGLT2i-mice (Fig 2G). The NE decline in BAT after treatment with α-MT, a catecholamine synthesis inhibitor, was significantly smaller in SGLT2i-mice (Fig 2G), indicating that acute SGLT2i treatment decreases both NE production and NE turnover. Therefore, we next examined the neural activity in the rRPa (Fig 2H), which contains the putative sympathetic premotor neurons responsible for thermogenesis in BAT [15]. Expressions of *c-fos*, a molecular marker of neuronal activation, in the rRPa were significantly decreased compared with control-mice 18 hours after dapagliflozin administration (Fig 2I). In addition, the number of c-fos positive neurons in the rRPa of SGLT2i-mice was significantly reduced as compared with those of control-mice (Fig 2J and 2K). These results supported the notion that sympathetic activity to BAT was suppressed. In contrast, plasma leptin (Fig 2L), glucagon (Fig 2M), NE (Fig 2N) and epinephrine (Fig 2O) levels did not differ between the two groups of mice. Collectively, these results suggest that dapagliflozin treatment acutely suppresses BAT activity via decreased sympathetic nerve activity, rather than via regulations mediated by these humoral factors.

### Dapagliflozin enhances hepatic gluconeogenesis and glycogenolysis in mice

**Fig 3:**
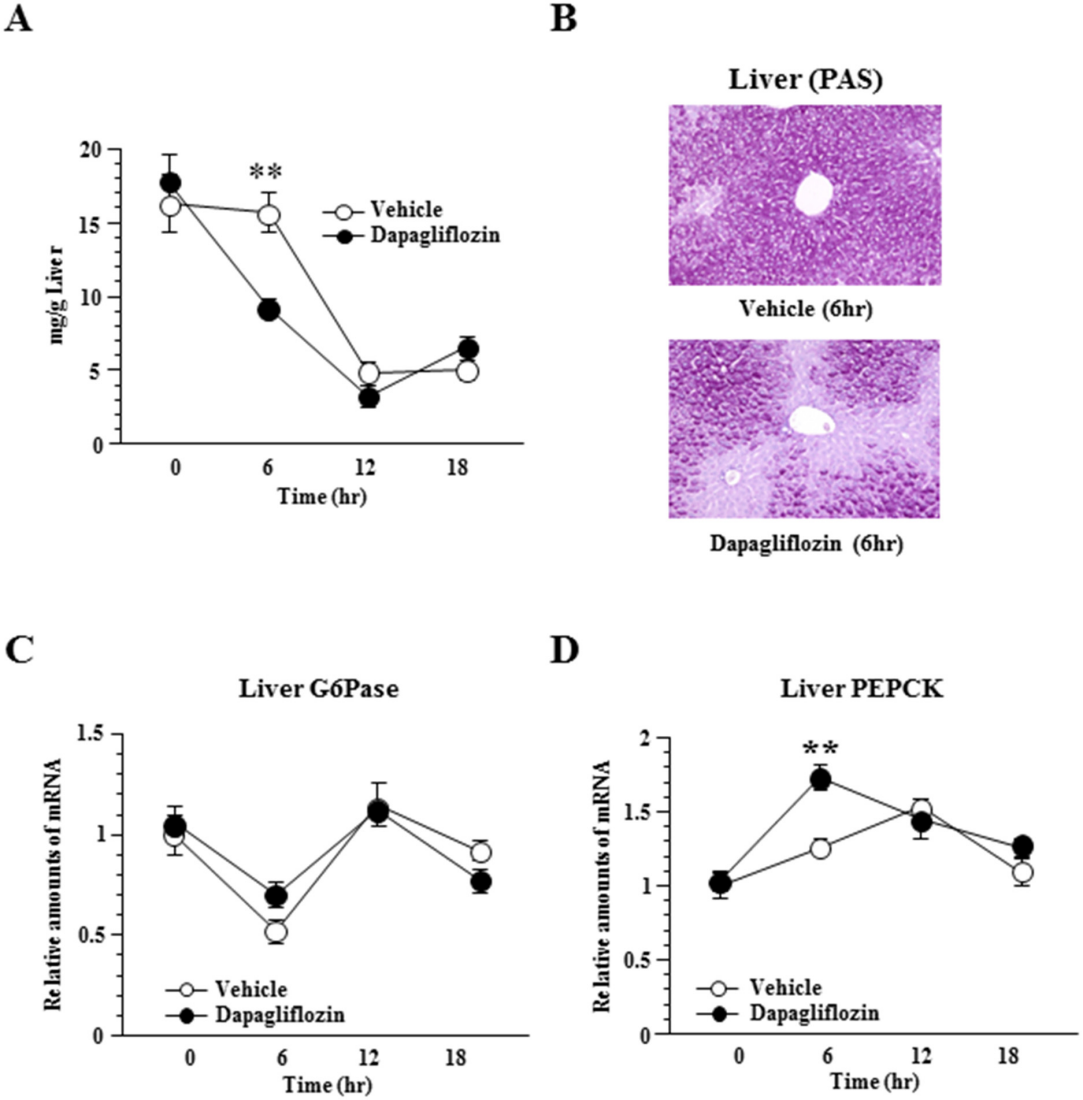
Dapagliflozin enhances hepatic gluconeogenesis and glycogenolysis. Hepatic glycogen accumulation (A), and histological findings with periodic acid-Schiff (PAS) staining of the liver 6 hours after dapagliflozin administration (B). Relative amounts of G6Pase (C) and PEPCK (D) mRNA in the liver. Values shown are means ±SEM (n = 5–7 per group). Significance as compared to control-mice is indicated (**P < 0.01).

In previous studies including ours, metabolic alterations in the liver were found to modulate sympathetic nerve activities to BAT [10,11] and WAT [16,17]. Therefore, we hypothesized that alteration of hepatic glucose metabolism is involved in suppression of sympathetic nerve activities to BAT in SGLT2i-mice. Dapagliflozin more acutely decreased hepatic glycogen contents than the vehicle. As shown in Fig 3A and 3B, glycogen contents in the livers of SGLT2i-mice were significantly lower after 6 hours but the differences disappeared after 12 hours. In addition, although expressions of glucose-6-phosphatase *(G6Pase)* mRNA did not differ between SGLT2i- and control-mice (Fig 3C), hepatic expression of phosphoenolpyruvate carboxykinase (*PEPCK*) was increased and reached a significantly higher level in SGLT2i- than in control-mice 6 hours after dapagliflozin administration (Fig 3D). At 12 hours after administration, however, *PEPCK* expression in control-mice was also increased to levels similar to those of SGLT2i-mice.These findings suggest both gluconeogenesis and glycogenolysis to be acutely enhanced by dapagliflozin treatment. Considering that suppressions of BAT activity, oxygen consumption and *c-fos* in the rRPa were detected 18 hours after administration, acute changes in hepatic glucose metabolism might trigger the subsequent attenuation of sympathetic activity from the rRPa to BAT.

### Common hepatic branch vagotomy attenuated suppression of BAT thermogenesis induced by dapagliflozin administration in mice

**Fig 4:**
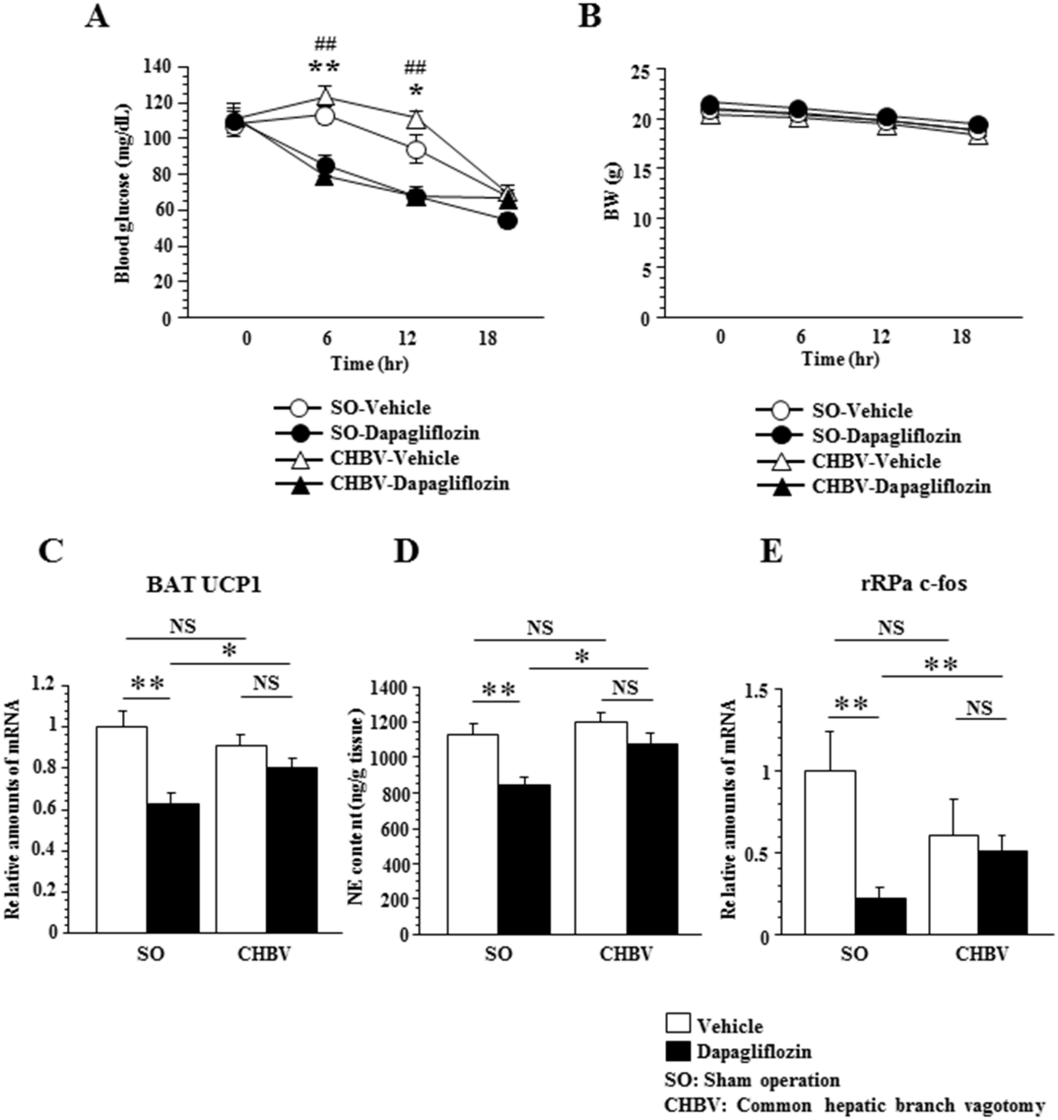
Common hepatic branch vagotomy attenuated suppression of BAT thermogenesis induced by dapagliflozin administration. Plasma blood glucose (A) and BW (B) after dapagliflozin administration (n = 6–7 per group). Significance is indicated **(P < 0.01), *(P < 0.05) in SO-vehicle-mice versus SO-dapagliflozin-mice, ##(P < 0.01) in CHBV-vehicle-mice versus CHBV -dapagliflozin-mice. Relative amounts of UCP1 mRNA in BAT (C), NE content in BAT (D) (n = 6–7 per group) and relative amounts of c-fos mRNA in the rRPa (E) (n = 5–7 per group) 18 hours after dapagliflozin administration. Values shown are means ±SEM. Significance is indicated (**P < 0.01, *P < 0.05). NS = not significant.

We previously demonstrated that alterations in hepatic glucose metabolism contribute to BAT thermogenesis via a neuronal network, i.e. the liver-vagal nerve-brain-sympathetic nerve-BAT axis [10]. Therefore, to examine the role of the common hepatic vagal branch in the dapagliflozin-induced suppression of rRPa-sympathetic nerve-BAT activity, we dissected this branch. On the 7th day after CHBV or sham operation (SO), we administered either dapagliflozin or vehicle to mice in each group. CHBV did not affect fasting blood glucose levels or body weights in either SGLT2i- or control-mice (Fig 4A and 4B).

Intriguingly, however, CHBV significantly attenuated the reduction in BAT *UCP1* expression to levels that did not reach statistical significance (Fig 4C) and reduced the BAT NE contents (Fig 4D) in SGLT2i-mice 18 hours after dapagliflozin administration. Furthermore, while dapagliflozin induction significantly decreased *c-fos* expression in the rRPa of SO mice, this effect was reversed by CHBV (Fig 4E). These results indicate that neuronal signals through common hepatic vagal branch contribute to dapagliflozin-induced suppression of thermogenesis via reduced sympathetic transmission from the rRPa to BAT.

## Discussion

To the best of our knowledge, this is the first demonstration that SGLT2i treatment acutely reduces BAT thermogenesis, leading to suppression of systemic oxygen consumption. On the other hand, it was previously reported that SGLT2i treatment induced hyperphagia but did not affect oxygen consumption in *ad libitum* fed rats [6,18]. Since food intake enhances BAT thermogenesis, a process known as diet-induced thermogenesis [19], the reduction of BAT thermogenesis in response to SGLT2i treatment might be compensated under the experimental conditions of these studies. To eliminate the effects of food intake on BAT thermogenesis, therefore, we selected the condition of food deprivation after dapagliflozin administration. This allowed us to detect an acute reduction in oxygen consumption after SGLT2i administration. As with the negative energy balance induced by SGLT2i treatment, fasting reduces systemic oxygen consumption [20]. Taken together, these observations suggest that suppression of BAT thermogenesis may work in negative energy balance states to maintain energy stores. However, it remains to be elucidated whether this newly-recognized mechanism whereby SGLT2 inhibition acutely reduces BAT thermogenesis also functions under conditions of chronic SGLT2i treatment.

It should be noted that CHBV attenuated the suppression of BAT thermogenesis induced by dapagliflozin administration. Dissecting the common hepatic vagal branch denervates not only the liver, but also certain parts of the distal stomach and duodenum [21]. Therefore, in addition to the liver, the gastric tract including the duodenum would also be a candidate for the origin of neural signals induced by SGLT2i treatment. In addition, CHBV dissects both efferent and afferent fibers contained in the vagal branch. Therefore, it cannot be ruled out that efferent vagal signals through the common hepatic vagal branch are involved in the BAT thermogenesis suppression observed in this study. However, SGLT2i does not reportedly affect carbohydrate metabolism in the duodenum. For instance, ipragliflozin, another highly selective SGLT2i, did not affect sucrose, maltose, glucose or fructose contents of the upper small intestine in mice [22]. Furthermore, since SGLT2 is not expressed in the gastrointestinal tract [23], direct effects of dapagliflozin on the gastrointestinal tracts are unlikely. In contrast, the glycogen content in the liver was markedly affected by dapagliflozin treatment prior to the alterations in BAT. The hepatoportal glucose sensing mechanism reportedly stimulates glucose utilization in peripheral tissue via a neural network consisting of afferent and efferent vagal nerves [24]. Furthermore, we previously showed that adenovirus-mediated expression of peroxisome proliferator-activated receptor γ2 in the liver induced acute hepatic steatosis, and increased BAT thermogenesis through afferent vagal signals originating in the liver [11]. We further showed that activation of glucokinase enhances hepatic glycogen accumulation, leading to suppression of BAT thermogenesis via regulation of sympathetic nerve activity [10]. These reports suggest that the liver-brain-BAT axis responding to hepatic glycogen storage levels contributes to maintaining an appropriate systemic energy state. The present study additionally suggests suppression of BAT thermogenesis via the common hepatic vagal branch to follow severe hepatic glycogen deprivation induced by SGLT2i treatment. Moreover, CHBV reversed rRPa *c-fos* downregulation induced by dapagliflozin treatment, indicating that neuronal signals through the common hepatic vagal branch affect the responses in the rRPa. Taking these findings together, neuronal signals from the liver, rather than those from the duodenum or efferent signals through the common hepatic vagal branch, appear to play a major role in dapagliflozin-induced suppression of BAT thermogenesis. Taking the results of the present and prior studies together, the liver may play an important role in triggering neural signals which in turn regulate systemic energy expenditure.

In this study, we demonstrated that SGLT2i treatment, which induces a negative energy balance state, acutely suppresses BAT thermogenesis and energy expenditure via neural signals. As to the possible physiological relevance of this mechanism, it may have functioned in favor of the survival of individuals during periods when stable food supplies from natural environments were not available. However, once an individual becomes obese in modern society, this system might make it difficult to reverse this state even when caloric intake is acutely restricted. This neuronal network may play an important role in body weight homeostasis and constitute a clinical target for the prevention and treatment of obesity.

## References

[1] Vallon V (2015) The mechanisms and therapeutic potential of SGLT2 inhibitors in diabetes mellitus. Annu Rev Med 66: 255–270. 10.1146/annurev-med-051013-110046 25341005

[2] Ferrannini E, Solini A (2012) SGLT2 inhibition in diabetes mellitus: rationale and clinical prospects. Nat Rev Endocrinol 8: 495–502. 10.1038/nrendo.2011.243 22310849

[3] Bailey CJ (2011) Renal glucose reabsorption inhibitors to treat diabetes. Trends Pharmacol Sci 32: 63–71. 10.1016/j.tips.2010.11.011 21211857

[4] List JF, Woo V, Morales E, Tang W, Fiedorek FT (2009) Sodium-glucose cotransport inhibition with dapagliflozin in type 2 diabetes. Diabetes Care 32: 650–657. 10.2337/dc08-1863 19114612 PMC2660449

[5] Vickers SP, Cheetham SC, Headland KR, Dickinson K, Grempler R, et al (2014) Combination of the sodium-glucose cotransporter-2 inhibitor empagliflozin with orlistat or sibutramine further improves the body-weight reduction and glucose homeostasis of obese rats fed a cafeteria diet. Diabetes Metab Syndr Obes 7: 265–275. 10.2147/DMSO.S58786 25061325 PMC4085306

[6] Devenny JJ, Godonis HE, Harvey SJ, Rooney S, Cullen MJ, et al (2012) Weight loss induced by chronic dapagliflozin treatment is attenuated by compensatory hyperphagia in diet-induced obese (DIO) rats. Obesity (Silver Spring) 20: 1645–1652. 22402735 10.1038/oby.2012.59

[7] Nagata T, Fukuzawa T, Takeda M, Fukazawa M, Mori T, et al (2013) Tofogliflozin, a novel sodium-glucose co-transporter 2 inhibitor, improves renal and pancreatic function in db/db mice. Br J Pharmacol 170: 519–531. 10.1111/bph.12269 23751087 PMC3791991

[8] Ferrannini G, Hach T, Crowe S, Sanghvi A, Hall KD, et al (2015) Energy Balance After Sodium-Glucose Cotransporter 2 Inhibition. Diabetes Care 38: 1730–1735. 10.2337/dc15-0355 26180105 PMC4542276

[9] Takahashi K, Yamada T, Tsukita S, Kaneko K, Shirai Y, et al (2013) Chronic mild stress alters circadian expressions of molecular clock genes in the liver. Am J Physiol Endocrinol Metab 304: E301–309. 10.1152/ajpendo.00388.2012 23211520

[10] Tsukita S, Yamada T, Uno K, Takahashi K, Kaneko K, et al (2012) Hepatic glucokinase modulates obesity predisposition by regulating BAT thermogenesis via neural signals. Cell Metab 16: 825–832. 10.1016/j.cmet.2012.11.006 23217261

[11] Uno K, Katagiri H, Yamada T, Ishigaki Y, Ogihara T, et al (2006) Neuronal pathway from the liver modulates energy expenditure and systemic insulin sensitivity. Science 312: 1656–1659. 16778057 10.1126/science.1126010

[12] Desautels M, Dulos RA (1988) Effects of repeated cycles of fasting-refeeding on brown adipose tissue composition in mice. Am J Physiol 255: E120–128. 3407768 10.1152/ajpendo.1988.255.2.E120

[13] Morrison SF, Nakamura K, Madden CJ (2008) Central control of thermogenesis in mammals. Exp Physiol 93: 773–797. 10.1113/expphysiol.2007.041848 18469069 PMC2496891

[14] Young JB, Saville E, Rothwell NJ, Stock MJ, Landsberg L (1982) Effect of diet and cold exposure on norepinephrine turnover in brown adipose tissue of the rat. J Clin Invest 69: 1061–1071. 7068845 10.1172/JCI110541 PMC370170

[15] Nakamura K, Matsumura K, Kaneko T, Kobayashi S, Katoh H, et al (2002) The rostral raphe pallidus nucleus mediates pyrogenic transmission from the preoptic area. J Neurosci 22: 4600–4610. 12040067 10.1523/JNEUROSCI.22-11-04600.2002 PMC6758794

[16] Izumida Y, Yahagi N, Takeuchi Y, Nishi M, Shikama A, et al (2013) Glycogen shortage during fasting triggers liver-brain-adipose neurocircuitry to facilitate fat utilization. Nat Commun 4: 2316 10.1038/ncomms3316 23939267 PMC3753545

[17] Uno K, Yamada T, Ishigaki Y, Imai J, Hasegawa Y, et al (2015) A hepatic amino acid/mTOR/S6K-dependent signalling pathway modulates systemic lipid metabolism via neuronal signals. Nat Commun 6: 7940 10.1038/ncomms8940 26268630 PMC4557134

[18] Yokono M, Takasu T, Hayashizaki Y, Mitsuoka K, Kihara R, et al (2014) SGLT2 selective inhibitor ipragliflozin reduces body fat mass by increasing fatty acid oxidation in high-fat diet-induced obese rats. Eur J Pharmacol 727: 66–74. 10.1016/j.ejphar.2014.01.040 24486393

[19] van Marken Lichtenbelt WD, Schrauwen P (2011) Implications of nonshivering thermogenesis for energy balance regulation in humans. Am J Physiol Regul Integr Comp Physiol 301: R285–296. 10.1152/ajpregu.00652.2010 21490370

[20] Shibata H, Bukowiecki LJ (1987) Regulatory alterations of daily energy expenditure induced by fasting or overfeeding in unrestrained rats. J Appl Physiol (1985) 63: 465–470. 3477537 10.1152/jappl.1987.63.2.465

[21] Berthoud HR (2004) Anatomy and function of sensory hepatic nerves. Anat Rec A Discov Mol Cell Evol Biol 280: 827–835. 15382018 10.1002/ar.a.20088

[22] Tahara A, Kurosaki E, Yokono M, Yamajuku D, Kihara R, et al (2012) Pharmacological profile of ipragliflozin (ASP1941), a novel selective SGLT2 inhibitor, in vitro and in vivo. Naunyn Schmiedebergs Arch Pharmacol 385: 423–436. 10.1007/s00210-011-0713-z 22139434

[23] Chen LH, Leung PS (2013) Inhibition of the sodium glucose co-transporter-2: its beneficial action and potential combination therapy for type 2 diabetes mellitus. Diabetes Obes Metab 15: 392–402. 10.1111/dom.12064 23331516

[24] Niijima A (1988) The effect of endogenous sugar acids on the afferent discharge rate of the hepatic branch of the vagus nerve in the rat. Physiol Behav 44: 661–664. 2853388 10.1016/0031-9384(88)90332-0

